# Development of a prognostic scoring system for chronic myeloid leukemia in blast phase

**DOI:** 10.1038/s41375-026-02875-9

**Published:** 2026-02-19

**Authors:** Michael Lauseker, Tomasz Sacha, Hana Klamova, Julia Yakoleva, Elza Lomaia, Dragana Milojkovic, Christian Fabisch, Franck E. Nicolini, Ulla Olsson-Stromberg, Daniela Zackova, Krzysztof Lewandowski, Philipp Ernst, Fausto Castagnetti, Klaus Hirschbühl, Irena Zupan, Astghik Voskanyan, Aleksandra Gołos, Jochen J. Frietsch, Justyna Gil, Edgar Faber, Stefan W. Krause, Anne Parry, Susanne Saussele, Sonja Heibl, Georg-Nikolaus Franke, Luis Felipe Casado, Edyta Paczkowska, Holger Hebart, Matthias Eder, Peter Anhut, Karolin Trautmann-Grill, Elzbieta Szczepanek, Thomas Südhoff, Elzbieta Patkowska, Alexander Kiani, Thomas Ernst, Jane Apperley, Jiri Mayer, Andreas Hochhaus, Markus Pfirrmann, Annamaria Brioli

**Affiliations:** 1 Institut für Medizinische Informationsverarbeitung, Biometrie und Epidemiologie (IBE), Medizinische Fakultät, Ludwig-Maximilians-Universität München, Munich, Germany; 2 Jagiellonian University Medical College, Department of Hematology, Krakow, Poland; 3 Institute of Hematology and Blood Transfusion, Prague, Czech Republic; 4 RM Gorbacheva Research Institute, Pavlov University, Saint Petersburg, Russia; 5 Research Department of Immuno-Oncology, Almazov National Medical Research Centre, Saint Petersburg, Russian Federation; 6 Hammersmith Hospital, Imperial College NHS Trust, London, UK; 7 Klinik für Innere Medizin II, Universitätsklinikum Jena, Jena, Germany; 8 Centre Léon Bérard, Hématology Department, Lyon, France; 9 CRCL INSERM U1052, Lyon, France; 10 University Hospital Uppsala, Department of Hematology, Uppsala, Sweden; 11 Department of Internal Medicine, Hematology and Oncology, University Hospital Brno and Masaryk University, Brno, Czech Republic; 12 Department of Hematology & Bone Marrow Transplantation, Poznań University of Medical Sciences, Poznań, Poland; 13 IRCCS Azienda Ospedaliero-Universitaria di Bologna, Istituto di Ematologia “Seràgnoli”, Bologna, Italy; 14 Dipartimento di Medicina Specialistica, Diagnostica e Sperimentale, Università di Bologna, Bologna, Italy; 15 Hematology and Oncology, Faculty of Medicine, University of Augsburg, Augsburg, Germany; 16 Department of Haematology, University Medical Centre Ljubljana, Ljubljana, Slovenia; 17 Medical Faculty, University of Ljubljana, Ljubljana, Slovenia; 18 Hematology Center after Prof. R. Yeolyan, Yerevan, Armenia; 19 Hematooncology Department, Copernicus Memorial Hospital, Lodz, Poland; 20 Medizinischen Klinik und Poliklinik II, Uniklinikum Würzburg, Würzburg, Germany; 21 Oncology Centre of the Podkarpackie Province, Department of Hematooncology, Brzozow, Poland; 22 Department of Hemato-Oncology, University Hospital Olomouc, Faculty of Medicine and Dentistry, Palacky University in Olomouc, Olomouc, Czech Republic; 23 Uniklinik Erlangen, Medizinische Klinik 5, Erlangen, Germany; 24 Centre Hospitalier Annecy Genevois, Annecy, France; 25 III. Med. Klinik, Med. Fakultät Mannheim, Universität Heidelberg, Mannheim, Germany; 26 Abteilung für Innere Medizin IV, Klinikum Wels-Grieskirchen, Wels, Austria; 27 University of Leipzig Medical Center, Department of Hematology, Cellular Therapy, Hemostaseology and Infectious Diseases, Leipzig, Germany; 28 Servicio de Hematología, Hospital General Universitario de Toledo, Toledo, Spain; 29 Department of General Pathology, Pomeranian Medical University in Szczecin, Szczecin, Poland; 30 Zentrum für Innere Medizin, Hämatologie/Onkologie, Stauferklinikum Schwäbisch-Gmünd, Mutlangen, Germany; 31 Klinik für Hämatologie, Hämostaseologie, Onkologie und Stammzelltransplantation, Medizinische Hochschule Hannover, Hannover, Germany; 32 Onkologische Schwerpunktpraxis Anhut, Kronach, Germany; 33 Medizinische Klinik I, Universitätsklinikum Carl Gustav Carus, TU Dresden, Dresden, Germany; 34 Klinikum Passau, Klinik für Onkologie, Hämatologie und Palliativmedizin, Passau, Germany; 35 Hematology Department, Institute of Hematology and Transfusion Medicine, Warsaw, Poland; 36 Medizinische Klinik IV, Klinikum Bayreuth GmbH, Bayreuth, and Comprehensive Cancer Center Erlangen-EMN, Bayreuth, Germany; 37 Centre for Haematology, Imperial College NHS Trust, London, UK; 38 Klinik und Poliklinik für Innere Medizin C, Hämatologie und Onkologie, Universitätsmedizin Greifswald, Greifswald, Germany

**Keywords:** Chronic myeloid leukaemia, Risk factors

## Abstract

Currently there is no established prognostic scoring system for patients with chronic myeloid leukemia (CML) in blast phase (BP). This study aimed to identify prognostic factors of CML-BP in a large cohort of prospectively and retrospectively collected patients and to develop a readily available prognostic scoring system at the onset of BP to enable future comparison between different trials and series. The analyses were based on 275 patients from thirteen countries, collected within the European LeukemiaNet Blast Phase Registry with a median observation time of 45 months and a median OS of 18.9 months. A Cox proportional hazards model for overall survival (OS) was employed, missing values were imputed. The study identified six independent prognostic factors: blast percentage, platelet count, age (all at onset of CML-BP), immunophenotype of BP, extramedullary disease, and previous history of CML. The low-risk group, encompassing 14% of patients, had a median OS of 97 months, the intermediate-risk group (59% of patients) of 22 months, and the high-risk group (27% of patients) of 9 months. Cross-validation demonstrated a good performance of the score, although external validation is strongly recommended. Despite the inherent limitations of registry data, the findings offer robust insights into prognostic factors for CML-BP.

## Introduction

In contrast to chronic phase (CP), chronic myeloid leukemia (CML) in blast phase (BP) still has a dismal prognosis with a median survival of 1–2 years [1]. The implementation of tyrosine kinase inhibitors (TKI) in the treatment of CP-CML has drastically reduced the incidence of BP, however, for those patients developing or being diagnosed in BP, prognosis did not substantially improve compared to the interferon era and before [2–5].

As opposed to CP-CML [6–10], evidence for prognostic factors of CML-BP remains scarce, largely due to its rarity. This renders comparisons between different patient series and interpretation of treatment outcomes difficult. The only large study investigating prognostic factors of CML-BP is the work by Jain et al. [11], who identified prognostic factors in 477 patients from a single center in the United States who developed CML-BP between 1997 and 2016. In this population myeloid immunophenotype, age ≥58 years, LDH ≥ 1227 IU/L, platelet count <102,000/μL, blast phase from CP / accelerated phase (AP) and presence of chromosome 15 aberrations were adverse prognostic for overall survival (OS). Based on data from a cohort of 51 patients collected between 1988 to 2013 in Mexico, Pérez-Jacobo et al. [12] identified lymphoid BP to be prognostically favorable and determined age, hemoglobin and complex karyotype as negative prognostic factors. In both cases, BP has been defined according to the WHO classification of 2008 [13], which defines a blast crisis as at least 30% blasts in the peripheral blood (PB) or bone marrow (BM). In both studies there were patients who were not treated with a TKI in CP and so might not be representative of a patient in CML-BP today. A more recent small study of 11 patients also showed a trend towards improved outcome for lymphoid-BP [14].

Based on the EUTOS registry, some of the authors of this paper have previously determined prognostic factors for de novo advanced phase CML patients [15]. That study identified age, percentages of blasts and basophils, hemoglobin concentration and additional chromosomal aberrations (ACAs) as prognostic factors. However, while these patients were diagnosed in the years 2008 to 2012, more than 60% of the patients were not in BP but in what was at the time called AP with blast percentages of 15–29%.

In the specific situation of CML-BP, a predictive score—namely a score that is able to predict responses to different treatments and guide treatment choices—is currently not of particular interest, as, as we have previously shown [4], treatment is heterogeneous and based on the individual patient and disease characteristics. Allogeneic stem cell transplantation is widely considered the treatment of choice, if applicable, although details on induction therapy, the timing of transplant and management post-transplant remain unclear [16]. On the other hand, a prognostic score would enable the comparison of different data sets and could be used for patients’ stratification in a clinical trial.

The aim of the present study was to identify prognostic factors for CML-BP in a large cohort of prospectively and retrospectively collected patients and to develop a useful prognostic scoring system at diagnosis enabling future comparison between different trials and patient series.

## Patients

The European LeukemiaNet Blast Phase Registry contains data on CML-BP patients from 13 countries (Armenia, Austria, Czech Republic, France, Germany, Italy, Poland, Russia, Slovenia, Spain, Sweden, United Kingdom). All patients were diagnosed with CML-BP after the 1st of January 2015. Patient data were collected retrospectively for deceased patients and prospectively for all other patients. The project has been registered under ClinicalTrials.gov ID NCT03869502. Details on the registry have been published [4].

## Methods

This study follows the definition from the 2022 WHO classification [17] and considers CML a bi-phasic disease with only BP and CP, excluding AP.

### Statistical analysis

Patients were observed from onset of BP to death or end of follow-up, whichever came first. Surviving patients were censored at the last follow-up.

Kaplan-Meier curves and log-rank tests respectively Cox proportional hazard models for overall survival (OS) were employed to describe and compare survival. In the multiple proportional hazard models, model selection was conducted using likelihood ratio tests. Cut-offs for the score were determined using the minimal *p* value approach. As expected, due to the nature of the registry, some data were missing. On average, ten percent of the data were missing. Details on the pattern of missing data are described in the Supplementary Material. Missing values were addressed through 20-fold multiple imputation via fully conditional specification [18, 19]. For the imputation model, all available variables were used with the addition of outcome. Results were pooled applying Rubin’s rules [20]. Internal model validation was performed using five-fold cross-validation, Harrell’s C-index was calculated. For details on the methodology, please see the supplemental material. *p* values < 0.05 were considered as significant.

Data management was performed using SAS 9.4, analyses were done in R 4.2.2. Besides standard ones, the following packages were used: *mice* for multiple imputation [21] and *survmetrics* for the C- index.

### Modeling considerations

The multiple model included 17 factors: platelets, hemoglobin, total white blood cell counts, percentages of basophils, eosinophils, and blasts from PB, BM promyelocytes, age (all at onset of BP), immunophenotype, *BCR::ABL1* transcript type (typical vs. atypical), presence of high-risk additional cytogenetic abnormalities (ACAs) [22], presence of *BCR::ABL1* mutations, involvement of the central nervous system (CNS), extramedullary disease (EM), time between CML diagnosis and BP development, phase at CML diagnosis, and sex. These variables were selected based on clinical and biological rationale and based to their wide availability at every center, to ensure an easy application of the score. Certain variables, such as blasts in the bone marrow, were excluded due to their high correlation with other included variables (i.e. PB-blasts). Information on specific *BCR::ABL1* mutations and high-risk ACAs was available in the data. However, except complex karyotype, +Ph and +8, no single aberration was frequent enough to be analyzed separately. Similarly, individual *BCR::ABL1* mutations were too rare to allow meaningful statistical analysis. An additional aim of the modeling was to ensure that the resulting score could be practically applied at the time of BP diagnosis. Since CML may have been diagnosed several years earlier—and treatment in the chronic phase may have been administered by a different physician—we sought to minimize reliance on historical data. As such, only two variables from the time of CML diagnosis were retained: phase at diagnosis and time to BP onset, as these should be available for almost all patients.

Information on treatment was intentionally not included, as the detailed treatment strategy may be unknown at the time of BP diagnosis. A detailed discussion of this rationale is provided in the “Discussion” section. Nevertheless, to test the robustness of the model, sensitivity analyses including allogeneic transplantation were performed.

For the final model, the creation of up to three prognostic groups was permitted, requiring that each group contain at least 10% of the total patient cohort. This threshold ensured that subgrouping remained statistically meaningful and clinically practical.

### Ethics approval and consent to participate

The project was approved by the respective ethics committee in each center and was conducted in accordance with the principles of good clinical practice and the Declaration of Helsinki. All living patients included in the registry provided written informed consent. For patients who were already deceased at the time of enrollment, informed consent was deemed not necessary by the ethics committee. In these cases, fewer personal data were collected to protect patients’ confidentiality.

## Results

### Sample

In total, 305 patients were included in the registry, of whom 275 met the inclusion and exclusion criteria and were deemed evaluable for the analysis. Thirty patients had to be excluded as they were not diagnosed between 2015 and 2022 (*n* = 11), not in BP (*n* = 1), had no informed consent (*n* = 2), had no baseline values to verify BP (*n* = 13) or had no reported outcome data (*n* = 3).

Patient characteristics are provided in Table 1. The majority of the patients (62.5%) were diagnosed in CP, had a myeloid immunophenotype (56.3%) and were male (59.6%). Atypical *BCR::ABL1* transcript types (10.2%), high-risk ACAs (31.6%), extramedullary disease (21.4%), involvement of central nervous system (CNS) (9.8%) and *BCR::ABL1* mutations (18.9%) occurred in a minority of patients, yet enough to consider them in the multiple regression model. When considering only patients diagnosed in CP, the median time to BP was 2.5 years.

**Table 1:** Variables considered as prognostic factors.

Variable		*N*	Percentage
Phase at diagnosis of CML	CP^a^	172	62.5%
	BP	103	37.5%
Immunophenotype	Myeloid	143	56.3%
	Lymphoid	92	36.2%
	Mixed	14	5.5%
	Megakaryoblastic	5	2.0%
Sex	Male	164	59.6%
	Female	111	40.4%
*BCR::ABL1* Transcript type	Typical transcript	158	89.8%
	Atypical transcript	18	10.2%
Presence of high-risk ACA	Yes	87	31.6%
	No	188	68.4%
Extramedullary disease	Yes	54	21.4%
	No	198	78.6%
Central nervous system involvement	Yes	24	9.8%
	No	220	90.2%
*BCR::ABL1* mutations	Yes	52	18.9%
	No	223	81.1%
Variable		*N*	Median (Range)
Age at onset of BP (years)		275	49 (18–86)
Leukocytes (PB, 10^9^/L)		264	46.4 (0.4–783.8)
Basophils (PB, %)		223	1 (0–50)
Eosinophils (PB, %)		221	1 (0–41)
Blasts (PB, %)		258	27 (0–98)
Platelets (PB, 10^9^/L)		255	97 (1.2–4800)
Hemoglobin (mmol/L)		254	6.4 (1.9–14.2)
Promyelocytes (bone marrow, %)		154	2 (0–73)
Time between diagnosis of CML and onset of BP (years)		275	0.63 (0–31.53)

*CML* chronic myeloid leukemia, *BP* blast phase, *CP* chronic phase, *PB* peripheral blood.

^a^This study follows the 2022 WHO definition [17] and considers CML a bi-phasic disease.

Median observation time in the cohort was 45 months (range: 0.1–103 months). During the follow-up time, 170 patients died. The median survival from onset of BP was 18.9 months (95% confidence interval (CI): 16.8–26.1 months).

### Univariate analyses

**Fig. 1:**
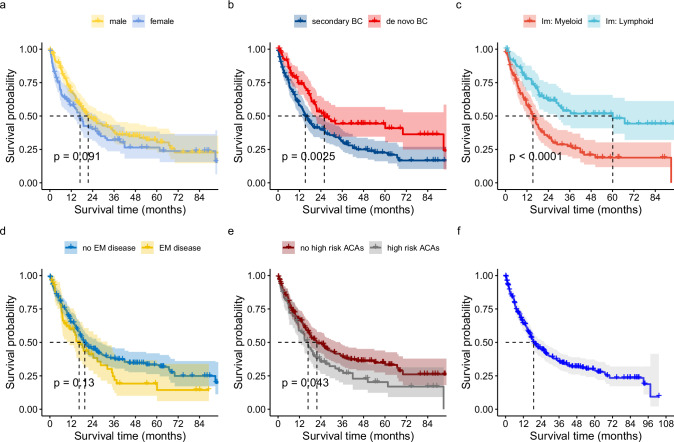
Mortality of CML-BP patients. Univariate survival according to sex (**a**), phase at diagnosis (**b**), immunophenotype (**c**), extramedullary disease (**d**), and high-risk ACAs (**e**) and overall survival in the complete sample (**f**).

At first the prognostic influence of the 17 candidate variables was analyzed univariately, using Kaplan-Meier curves and Cox models. At this stage, only available data were included, no imputation was performed. Males were found to have slightly better outcomes than females, yet the difference was not significant (Fig. 1a). As already shown [4], patients with de novo BP had a survival advantage compared to patients with secondary BP (HR: 0.61, 95% CI: 0.44–0.84, *p* = 0.025) (Fig. 1b). Significant differences between myeloid and lymphoid BP (HR: 2.15, 95% CI: 1.59–3.09, *p* < 0.001) (Fig. 1c) were confirmed. The few patients with megakaryoblastic (HR: 2.13, *p* = n.s.) and mixed (HR: 2.05, *p* = n.s.) BP showed survival probabilities more similar to myeloid BP than to lymphoid BP. Patients with extramedullary disease had a slightly worse prognosis, however, differences were not significant (Fig. 1d). Patients with high-risk ACAs at onset of BP had an inferior survival compared to those without (HR: 1.38, 95% CI: 1.01–1.89, *p* = 0.043) (Fig. 1e). The most frequent ACAs were complex karyotype (22.9%), +8 (13.5%) and +Ph (11.2%) (Supplementary Table 1). For the first two, it was possible to show a significant survival disadvantage (Supplementary Figs. 1 and 2). +Ph did not significantly affect survival of BP. Both, complex karyotype and +8 were more frequently observed, when CML was diagnosed in CP (26% and 16%) compared to diagnosis in BP (17% and 10%). No significant effect on survival was found for CNS involvement, *BCR::ABL1* mutations, or *BCR::ABL1* transcript type.

Regarding continuous covariates, PB blast percentage was significantly associated with survival (HR per percentage point: 1.007, 95% CI: 1.001–1.013, *p* = 0.026). However, categorizing blasts into three groups (<20%, 20–30%, ≥30%) did not yield significant differences, suggesting a linear relationship. This was further supported by a martingale residual analysis (see Supplementary Materials). As expected, age at BP onset was a strong predictor of survival. The effect was nonlinear; inclusion of a quadratic age term (age²/100) significantly improved model fit, indicating a steeper increase in hazard with advancing age (HR: 1.034, 95% CI: 1.025–1.044, *p* < 0.001). In patients diagnosed in CP, the time between diagnosis of CML and onset of BP had no significant influence on prognosis (HR per month 1.030 CI: 0.995–1.065 *p* = n.s.). Similarly, no significant prognostic effects were observed for white blood cell count, basophils, eosinophils, platelets, hemoglobin, and promyelocytes.

### Multiple regression model

**Fig. 2:**
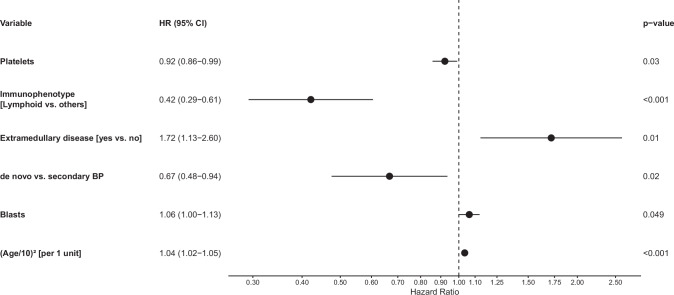
Multiple model. Final model for mortality showing the hazard ratios (HR) together with confidence intervals (CI) for the six covariates.

For the multiple regression analysis, imputed data were used. The full model containing all seventeen variables is shown in the Supplementary Material (Supplementary Table 2). Due to the rarity of megakaryoblastic and mixed immunophenotypes, and the similarity of their regression coefficients to those of the myeloid subtype, these three groups were combined for analysis. To facilitate interpretation, certain variables were transformed: platelets per 100 ×109/L, blast percentage per 10% and age. After performing variable selection, the final model retained six variables (Fig. 2): platelets at onset of BP, phase at diagnosis, immunophenotype (dichotomized as lymphoid vs. others), extramedullary disease, PB blast percentage at onset of BP, age at onset of BP (in squared form). According to this model, favorable prognosis is associated with younger patients presenting with a peripheral blast percentage as low as possible, a platelet count as high as possible, absence of extramedullary disease, no previous history of CML and CML diagnosed in lymphoid BP.

In a subsequent step, interaction effects between disease phase at CML diagnosis (de novo vs. secondary BP) and the remaining covariates were analyzed. None of the interaction terms were statistically significant or clinically meaningful, suggesting that the prognostic effects of these variables are consistent across both de novo and secondary BP. From the final model, a prognostic score was derived using the following equation: 0.061 × (Blasts [%] / 10) – 0.082 × Platelets [per 100 × 109/L] + 0.034 × Age² / 100 – 0.405 [if de novo BP] – 0.865 [if lymphoid immunophenotype] + 0.541 [if extramedullary disease]

**Fig. 3:**
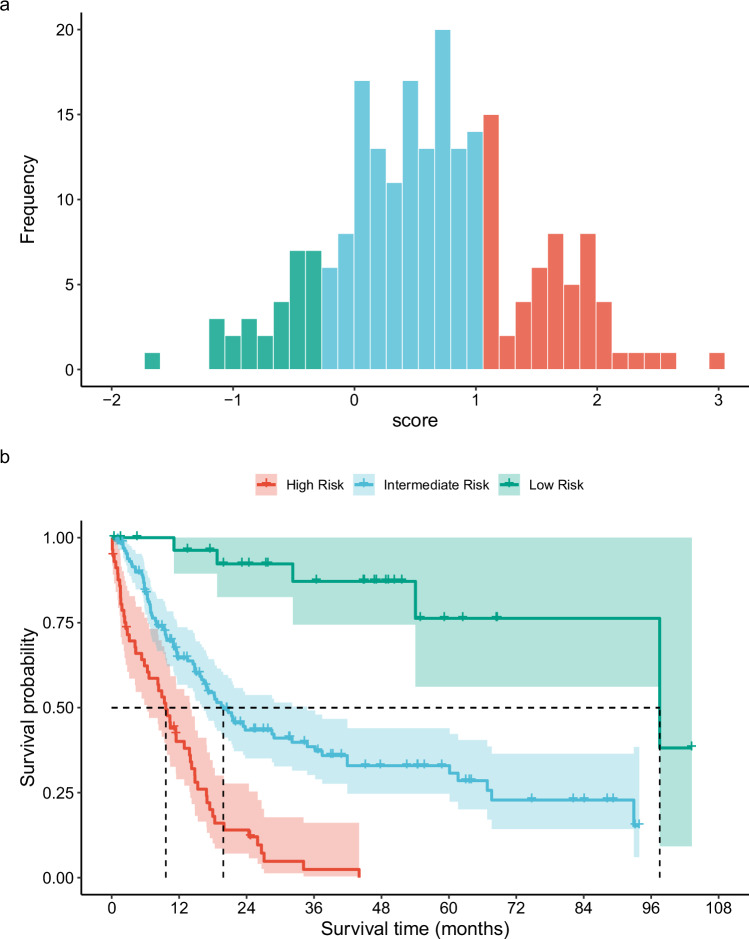
Categorization of the score. Histogram showing the distribution of the score (**a**) and survival in the combined set of all imputations according to the risk groups (**b**).

Based on this score, patients were stratified into three risk groups. A low-risk category was defined by a score less than −0.270 and contained 13.8% of the patients. The intermediate risk group lay between −0.270 and 1.059 and contained 59.4% of the patients. Patients with a score >1.059 were considered high risk; this group encompassed the remaining 26.8% (Fig. 3a). These groups differed significantly in terms of survival: the low-risk group showed a median survival of 98 months (95% CI: 98–NA), the intermediate-risk group a median survival of 22 months (95% CI: 17–31 months) and the high-risk group a median survival of 10 months (95% CI: 6–14 months) (Fig. 3b). Because risk group cut-offs were determined from pooled data across all imputations, no valid *p*-value can be reported for the survival comparison.

In the original data set without imputation, the low-risk group was predominantly composed of de novo BP cases (22/30). In contrast, the intermediate-risk (76/132) and especially the high-risk group (47/57) were mainly composed of cases of BP with a previous history of CML-CP. Few patients in the low-risk group had extramedullary disease (*n* = 3) or non-lymphoid immunophenotypes (*n* = 7), whereas the high-risk group contained only five patients with lymphoid immunophenotype.

### Cross-validation

Five-fold cross validation to evaluate the robustness of this model was performed. The variable selection process consistently retained all predictors from the final model, except for platelet count, which was selected in four out of five iterations. To measure model performance, Harrell’s concordance index (C-index) was used. The average C-index across the five folds was 0.70 for the full model and 0.66 for the stratified risk groups. As anticipated, these values were slightly lower than those obtained from the full imputed dataset, where the C-index reached 0.71 for the complete model and 0.68 for the risk groups.

### Sensitivity analyses

Several sensitivity analyses related to treatment were conducted. While these models are not intended for prognostic interpretation and most importantly do not correctly estimate a treatment effect, they offer insight into the robustness of the original model.

First, patients were censored at the time of transplantation and the final model was re-estimated. When censoring for transplantation, all coefficients in the final model retained the same direction as in the original analysis. However, for all coefficients except PB-blasts and phase at diagnosis of CML, the effects were slightly smaller. Notably, the effects of platelets (HR: 0.94, compared to 0.92) and extramedullary disease (HR: 1.51, compared to 1.72), were no longer significant on the 5% level. Subsequently, transplantation was incorporated as a time-dependent covariate. In this model, coefficients were even more similar to the original model. Again, attenuation to zero for all covariates except blasts and phase at diagnosis was observed. Of note, the age effect was reduced (HR: 1.01, compared to 1.04), though all *p* values remained significant at the 5% level.

Additionally, the inclusion of the three most frequent ACAs (complex karyotype, +8, +Ph) replacing the broader “high-risk ACA” category was explored. While significant effects for complex karyotypes and +8 were present in a univariate analysis, none of the three variables remained significant in the multiple model and none were retained in the final model. Finally, the model was re-estimated using bone marrow blasts instead of peripheral blood. Although both variables were correlated, no significant effect was found for bone marrow blasts. It seems likely that this is caused by the more homogeneous BM blast percentages.

## Discussion

While validated prognostic scores exist for CP-CML [6–9], no reliable prognostic score has been established for CML-BP. As previously demonstrated [4], CML-BP is a highly heterogeneous condition, that cannot be approached with a unifying strategy. Nevertheless, the ability to stratify patients using a simple, clinically applicable score would significantly advance research in this field—facilitating comparisons between patient cohorts and enhancing prognostic accuracy. To address this gap a comprehensive analysis of the European LeukemiaNet Blast Phase Registry was conducted with the goal of developing a score that will not only enhance further research but might help clinicians in defining the prognosis of CML-BP patients. The analysis identified six independent prognostic factors: percentage of blasts at diagnosis of CML-BP, platelet count, age at onset of CML-BP, immunophenotype, extramedullary disease, and phase at diagnosis of CML. Although differences between de novo and secondary BP were observed, the same set of prognostic factors applied to both subgroups. Based on this, a prognostic scoring system was developed with three well-differentiable groups: low, intermediate, and high risk.

Some of the prognostic factors are consistent with previous findings [11, 12] but because of different modeling—both previous studies used, e.g., dichotomization of continuous covariates—the results are not directly comparable. Moreover, Jain et al. included treatment information as well, which was deliberately avoided here for conceptual reasons (see below). The prognostic disadvantage of myeloid BP compared to lymphoid BP has been reported previously, although this effect in the multivariable model appeared more pronounced (HR 2.38 vs. 1.55 and 1.67, respectively). Consistent with our findings, Jain et al. [11] identified a high platelet count as a good prognostic factor and found de novo BP prognostically favorable in comparison to secondary BP, however they did not observe an effect of extramedullary disease. Pérez-Jacobo [12] highlighted hemoglobin as an important prognostic factor, which this study could not confirm.

Chromosomal aberrations showed variable impact across studies: Jain [11] identified chromosome 15 aberrations as prognostically unfavorable, whereas Pérez-Jacobo [12] emphasized complex karyotypes. In the present analysis, complex karyotypes and +8 were associated with poorer survival in univariate models, but not in the multivariable setting. One reason for these discrepancies might be that the presence of high-risk ACAs at onset of BP are more likely in those who progressed from CP than in those who did not, which appeared to be the stronger predictor—attenuating the effects of cytogenetic abnormalities once adjusted for. Additionally, CML-BP is a genetically more complex disease than CML-CP, where genetic factors other than cytogenetic abnormalities play a role. Unfortunately, at the time the registry was run, sequencing of CML-BP was not routinely performed, and data on mutations in genes other than *BCR::ABL1* were available only in a minority of patients and could not be included in the model. Aberrations of chromosome 3q26, a known risk factor in CML-CP, were present in only 8 patients and could not be analyzed separately. Future analysis in a larger dataset of patients should further evaluate the implication of additional mutations and gene aberrations in CML-BP. Atypical *BCR::ABL1* did not have an effect on survival. Again, this is likely due to the biology. Finally, age at onset of BP was—not surprisingly—identified as a prognostic factor in all studies, although modeled differently, thus limiting direct comparison.

In this work, myeloid immunophenotypes were grouped with mixed, and megakaryoblastic immunophenotypes. This choice was data-driven: both mixed and megakaryoblastic immunophenotypes were rare, and their outcomes closely resembled those of myeloid BP. More data might allow evaluating the impact of these rare phenotypes singularly in the future.

A key design choice is the exclusion of treatment from this model. This was based on several considerations: first, treatment of BP is both complex and highly individualized, influenced by drug availability, BP characteristics, patient comorbidities and previous toxicities and resistances. Patients included in the registry received a median of three different treatments with no consistent pattern [4]. Treatment regimens also varied substantially across centers and countries. Including treatment would have grossly oversimplified this complexity, and the sample size was insufficient to model all possible regimens. A formal statistical analysis of the effect of any treatment within a non-randomized, purely observational study such as this requires a different approach [23] and is not the focus of this analysis. Second, treatment decisions likely correlate with baseline prognostic factors, which introduces potential confounding. Including treatment in the model would risk bias by diluting the effects of these variables. Finally, our aim was to provide prognostic information available at BP onset—when treatment decisions have not yet been made.

Despite the exclusion of treatment from our model, still treatment heterogeneity could be seen as a limitation in our data. While sensitivity analyses accounting for treatment (e.g., censoring for transplantation or including it as a time-dependent variable) showed only minimal changes in regression coefficients, the possibility that favorable outcomes are partially driven by eligibility for a certain treatment, i.e. allogeneic stem cell transplantation, cannot entirely be excluded, and it seems likely that the plateau seen in survival curves reflects the success of allogeneic stem cell transplantation. From a statistical point of view, a homogeneously treated cohort of patients could have answered this question. However, due to the rarity and heterogeneity of BP, this is hardly feasible.

Conversely, the heterogeneity of the data, can also be seen as a strength, as the sample consists of patients from 13 different European countries. It enhances the generalizability of the findings across different healthcare systems as well as different treatment practices. Although external validation remains necessary, this diversity offers a solid foundation for broader application, potentially extending to non-European settings.

Another limitation of this work is the presence of missing data, a common challenge in observational studies. While multiple imputation is a well-established method, a complete data set would naturally be preferable.

It is important to note that this is not a predictive score (i.e. designed to assess the likely benefit of a particular therapeutic intervention), but a prognostic score, which provide estimates of survival independent of specific treatments. Our CML-BP score is designed to facilitate scientific comparison of patient cohorts and to possibly serve as a stratification tool in future clinical trials. It is important to emphasize that is not intended to guide treatment selection or to predict response to specific therapies. Nevertheless, it could help to characterize patient populations and might in the longer term support individualized care by e.g. enabling risk-adapted monitoring.

In summary, a prognostic score was developed and validated for patients with CML-BP based on routinely collected variables to determine prognostic significance. The tool can be used for future studies and assist physicians in assessing prognosis of patients with CML-BP, and lays the groundwork for ongoing collaborative assessment to determine important predictive factors.

## Supplementary information

Supplementary material

## Data Availability

Data are available from the authors upon reasonable request.
