# Supplementary material for: Development of a prognostic scoring system for chronic myeloid leukemia in blast phase
Source: Leukemia. 2026 Feb 19;40(4):751–8. doi: 10.1038/s41375-026-02875-9 (PMC13056570; doi:10.1038/s41375-026-02875-9)
Supplement: Supplementary file 1 — Supplementary material [file 41375_2026_2875_MOESM1_ESM.docx]

|  |  | Estimate | Standard error | HR | p |
| --- | --- | --- | --- | --- | --- |
| Leukocytes | per 1000 Gpt/L | -0.335 | 0.794 | 0.715 | 0.674 |
| Basophils | per 1% | 0.016 | 0.014 | 1.016 | 0.250 |
| Eosinophils | per 1% | -0.018 | 0.027 | 0.982 | 0.510 |
| Blasts | per 10% | 0.063 | 0.034 | 1.065 | 0.069 |
| Platelets | per 100 Gpt/L | -0.098 | 0.043 | 0.906 | 0.025 |
| Haemoglobin | per 1 mmol/L | -0.042 | 0.052 | 0.959 | 0.426 |
| Bone marrow promyelocytes | per 1% | 0.006 | 0.016 | 1.006 | 0.728 |
| Age²/100 | per 1 | 0.035 | 0.005 | 1.035 | < 0.001 |
| Time to BP | per 1 year | -0.009 | 0.019 | 0.991 | 0.643 |
| Sex | female vs male | 0.112 | 0.176 | 1.119 | 0.525 |
| Phase at diagnosis of CML | *de novo* vs. secondary BP | -0.412 | 0.241 | 0.662 | 0.090 |
| Immunophenotype | lymphoid vs. others | -0.869 | 0.197 | 0.419 | < 0.001 |
| Atypical transcripts | yes vs. no | 0.291 | 0.368 | 1.338 | 0.433 |
| *BCR::ABL1* mutation | yes vs. no | 0.256 | 0.214 | 1.292 | 0.233 |
| High risk ACA | yes vs. no | 0.102 | 0.172 | 1.108 | 0.551 |
| Extramedullary disease | yes vs. no | 0.599 | 0.259 | 1.820 | 0.023 |
| Central nervous system involvement | yes vs. no | 0.202 | 0.340 | 1.224 | 0.554 |

Supplementary table 1: Pooled multiple Cox model with all seventeen covariates

| ACA | N (yes) | Frequency | HR (yes vs. no) | 95% CI | p |
| --- | --- | --- | --- | --- | --- |
| +8 | 37 | 13.5% | 2.11 | [1.39; 3.19] | < 0.001 |
| +19 | 10 | 3.6% | 2.00 | [0.93; 4.29] | 0.075 |
| +21 | 11 | 4.0% | 1.24 | [0.61; 2.52] | 0.556 |
| +Ph | 31 | 11.2% | 1.51 | [0.97;2.35] | 0.067 |
| Chromosome 3 aberrations | 9 | 3.2% | 0.96 | [0.39; 2.34] | 0.929 |
| -7 / 7q | 17 | 6.2% | 1.19 | [0.66; 2.15] | 0.568 |
| i17q | 8 | 2.9% | 0.92 | [0.38; 2.25] | 0.854 |
| Complex Karyotype | 63 | 22.9% | 1.67 | [1.20; 2.34] | 0.003 |

Supplementary table 2: ACAs – frequencies and hazard ratios in a univariate model


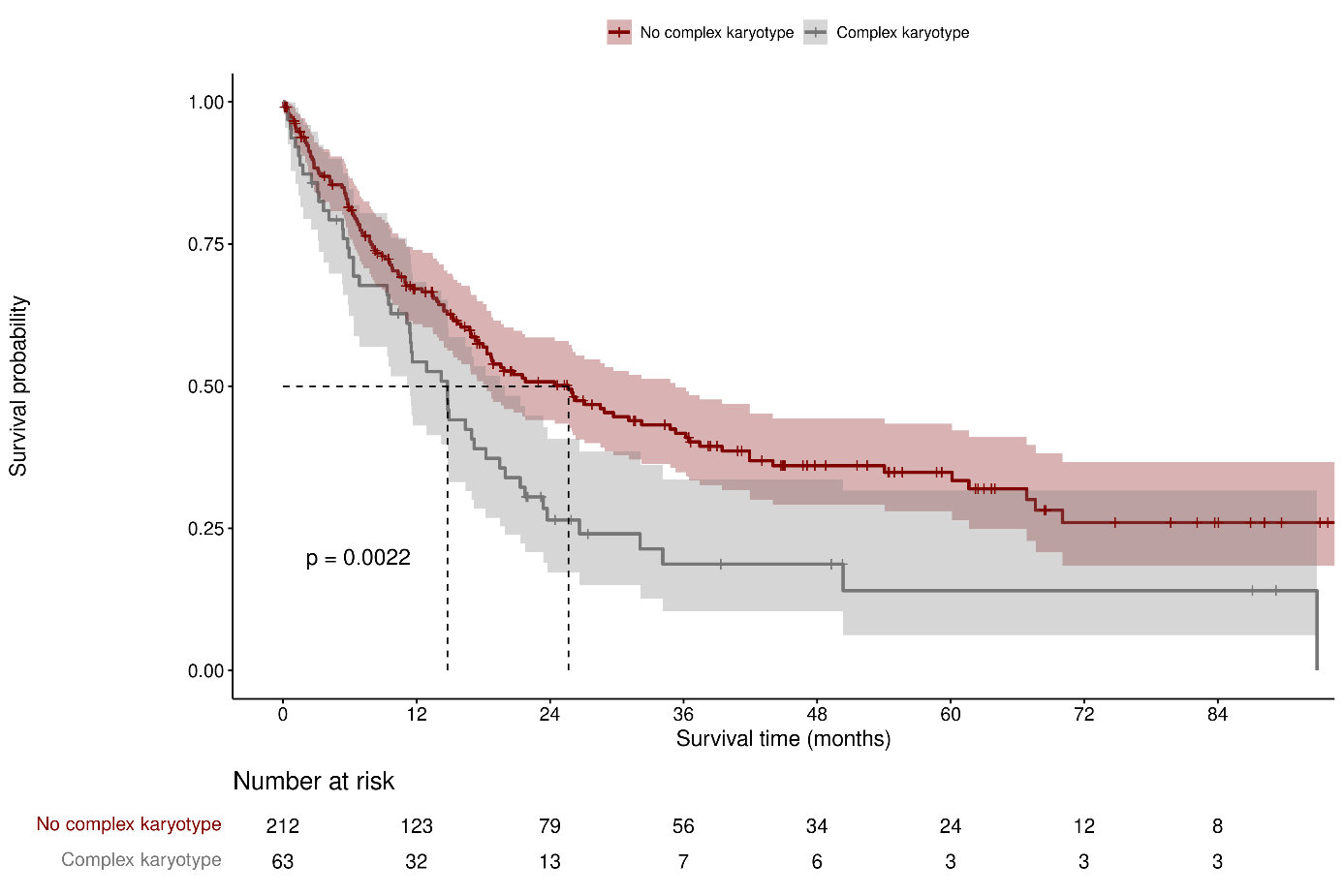


Supplementary Figure 1: Univariate survival according to complex karyotype


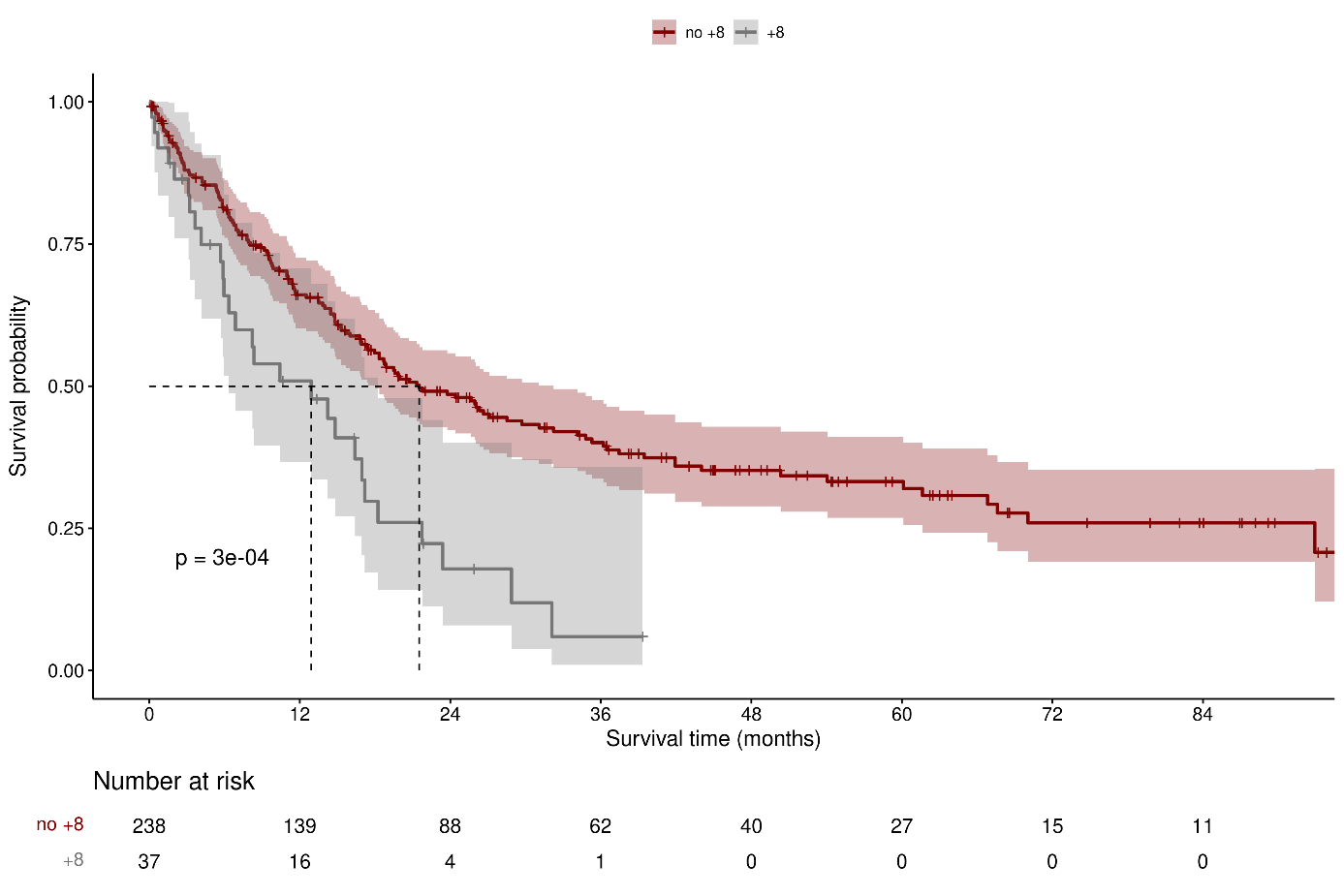


Supplementary Figure 2: Univariate survival according to presence of +8

Details on the statistical methodology

Missing values

Seventeen covariates were considered for model, of whom six were observed completely. Survival times were censored; however, no missingness was present in the dependent variable besides three observations that had only baseline data and were excluded from the analysis. The missingness pattern is shown below, the purple areas indicate missingness.


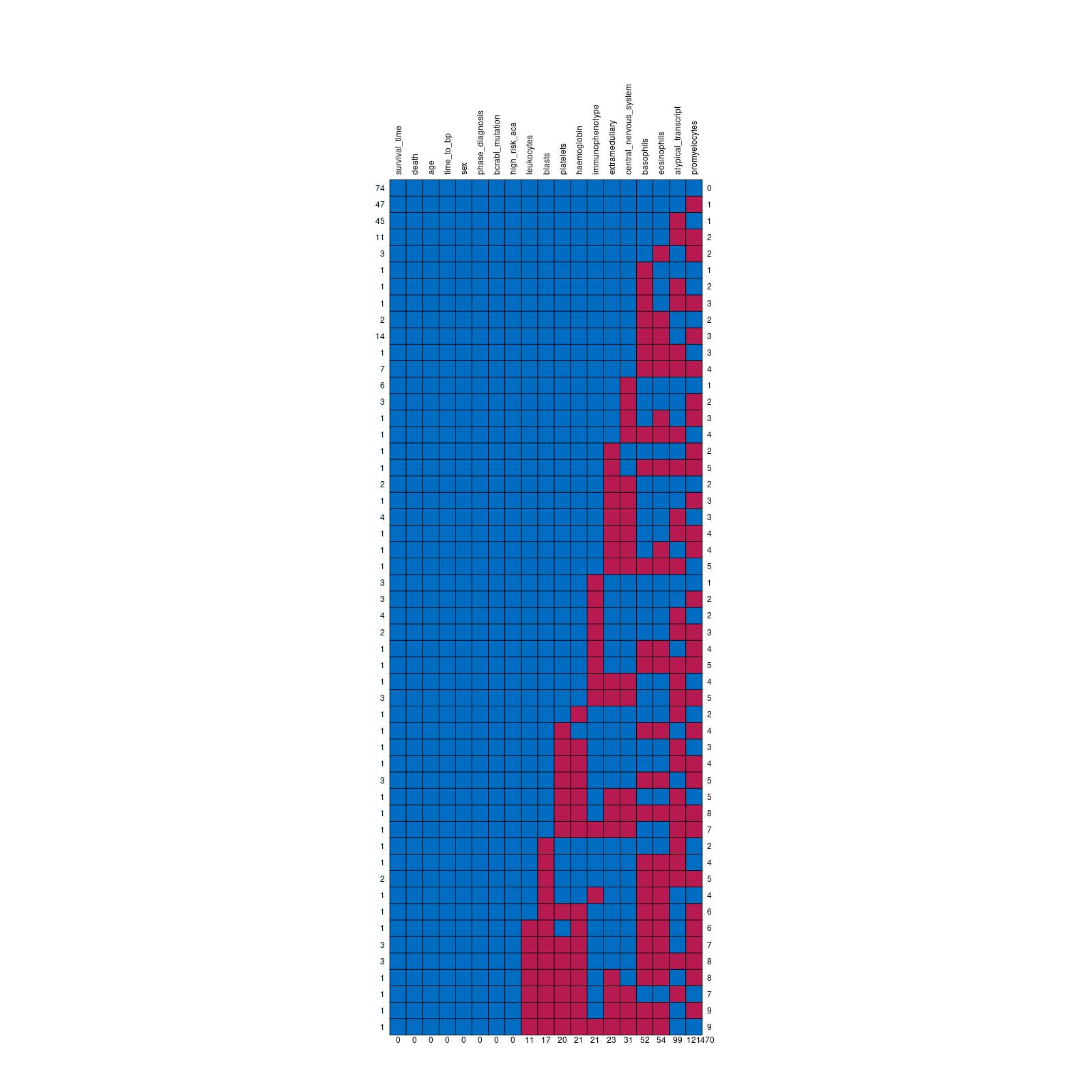


It can be seen that missingness occurs mainly in two variables, promyelocytes and atypical transcripts with 121 respectively 99 missing values each. 74 patients had no missing values at all, 47 patients had only missing promyelocytes etc.

For the imputation model, all variables available were used as predictors, including those that are not used for the model like e.g. bone marrow blasts or centre and also including the dependent variable. For binary variables like atypical transcript or presence of extramedullary disease, logistic regression was used as imputation method. For categorical variables like the immunophenotype, polytomous logistic regression was used and for continuous variables like basophils, imputation was done via predictive mean matching. Twenty complete data sets were generated.

Modelling

Within each of these twenty data sets, cox proportional hazards models were estimated. The form of the effect of continuous covariates like age was analysed beforehand in a univariate setting. For this, martingale residuals were analysed graphically. As a consequence, age² was included instead of age. A number of continuous variables like blasts were rescaled, by e.g. dividing by 10, to get an easier interpretation of the resulting hazard ratio.

Results of the twenty models were combined using Rubin’s rules. The final model was chosen by backward selection using the likelihood ratio test. Additionally, interaction effects between phase at diagnosis of CML (*de novo* BP vs. secondary BP) and continuous covariates were analysed. However, none of these improved the model. Statistically speaking, the inclusion of time between diagnosis of CML and onset of BP to the model is an interaction effect as well, as only secondary BP patients have a value other than zero.

The cut-offs were chosen from the final model containing the six covariates chosen (age, blasts, platelets, immunophenotype, extramedullary disease, phase at diagnosis of CML). To get unique cut-offs, data from the twenty imputed data sets were combined into one large data set with 5500 observations. Up to three groups were allowed, each group had to contain at least ten percent of the observations. The final cut-offs were determined using the minimal p-value approach.

Cross Validation

Five-fold cross validation was performed to assess the prognostic quality of the model. Multiple imputation and model selection were performed as described above in each of these five folds. Variable selection yielded similar results: Five out of six variables in our final model were selected in all cases. Platelets were chosen only in four out of five cases.
Harrell’s C was calculated separately and averaged over the twenty imputations and five folds.
This index lies between zero and one with the following interpretation:
For every pair of patients i and j (with i ≠ j), their risk scores and times-to-event are compared. If the risk score of i is larger than the risk score of j and the survival of I is shorter than the survival of j than this pair is considered concordant. If the risk score of i is smaller than the risk score of j and the survival of I is shorter than the survival of j than this pair is considered discordant. If due to censorings it can not be decided which of the patients lived longer, the pair is not counted. The C-index is calculated as the number of concordant pairs divided by the sum of concordant and discordant pairs. A value of 1 would indicate perfect prognostic ability while a value of 0.5 would be equivalent to a coin toss.
